# Metabolic Potential of the Gut Microbiome Is Significantly Impacted by Conditioning Regimen in Allogeneic Hematopoietic Stem Cell Transplantation Recipients

**DOI:** 10.3390/ijms231911115

**Published:** 2022-09-21

**Authors:** Mette Jørgensen, Jens C. Nørgaard, Emma E. Ilett, Ramtin Z. Marandi, Marc Noguera-Julian, Roger Paredes, Daniel D. Murray, Jens Lundgren, Cameron Ross MacPherson, Henrik Sengeløv

**Affiliations:** 1 CHIP Centre of Excellence, Rigshospitalet, 2100 Copenhagen, Denmark; 2 Department of Clinical Medicine, Faculty of Health and Medical Sciences, University of Copenhagen, 2200 Copenhagen, Denmark; 3 Institut de Recerca de la SIDA–IrsiCaixa, Hospital Universitari Germans Trias i Pujol, 08916 Badalona, Spain; 4 Centre for Health and Social Care Research (CESS), Faculty of Medicine, University of Vic—Central University of Catalonia (UVic—UCC), 08500 Vic, Spain; 5 Infectious Diseases Department, Hospital Universitari Germans Trias i Pujol, 08916 Badalona, Spain; 6 Department of Infectious Diseases, Rigshospitalet, 2100 Copenhagen, Denmark; 7 Department of Haematology, Rigshospitalet, 2100 Copenhagen, Denmark

**Keywords:** allogeneic hematopoietic stem cell transplantation (aHSCT), conditioning regimen, metabolic potential, gut microbiome, whole genome sequencing

## Abstract

Allogeneic hematopoietic stem cell transplantation (aHSCT) is a putative curative treatment for malignant hematologic disorders. During transplantation, the immune system is suppressed/eradicated through a conditioning regimen (non-myeloablative or myeloablative) and replaced with a donor immune system. In our previous study, we showed changes in gut taxonomic profiles and a decrease in bacterial diversity post-transplant. In this study, we expand the cohort with 114 patients and focus on the impact of the conditioning regimens on taxonomic features and the metabolic functions of the gut bacteria. This is, to our knowledge, the first study to examine the metabolic potential of the gut microbiome in this patient group. Adult aHSCT recipients with shotgun sequenced stool samples collected day −30 to +28 relative to aHSCT were included. One sample was selected per patient per period: pre-aHSCT (day −30–0) and post-aHSCT (day 1–28). In total, 254 patients and 365 samples were included. Species richness, alpha diversity, gene richness and metabolic richness were all lower post-aHSCT than pre-aHSCT and the decline was more pronounced for the myeloablative group. The myeloablative group showed a decline in 36 genera and an increase in 15 genera. For the non-myeloablative group, 30 genera decreased and 16 increased with lower fold changes than observed in the myeloablative group. For the myeloablative group, 32 bacterial metabolic functions decreased, and one function increased. For the non-myeloablative group, three functions decreased, and two functions increased. Hence, the changes in taxonomy post-aHSCT caused a profound decline in bacterial metabolic functions especially in the myeloablative group, thus providing new evidence for associations of myeloablative conditioning and gut dysbiosis from a functional perspective**.**

## 1. Introduction

Allogeneic hematopoietic stem cell transplantation (aHSCT) is a putative curative treatment for hematologic malignancies and other hematologic disorders. It involves suppression or eradication of the immune system through a conditioning regimen and replacement of the immune system with a new donor immune system. Conditioning regimens consist of chemotherapy with or without radiation and/or antibiotics. Conditioning regimens differ from hospital to hospital, but in general they are divided into intensive myeloablative and less intensive non-myeloablative regimens. During and after transplantations, life-threatening complications can occur, including acute graft versus host disease (aGvHD), infections and neutropenic fever. Several complications of aHSCT have been associated with the microbiome [1]. It is well established that the diversity of the microbiome declines post-transplant [2,3,4,5,6,7] and a lower diversity is associated with a higher risk of aGvHD and a lower rate of survival [3,4,6,8,9,10,11,12,13,14]. It has been shown that in many patients Enterococcus becomes the dominant species after transplantation and the expansion of Enterococcus has been linked to higher aGvHD risk [3,5,6,11,15]. It is to a lesser extent known if and how the intensity of the regimen is associated with the impact on the microbiome. However, a few studies indicate that the intensity of the conditioning regimen is associated with the species diversity, and gene richness [3,16]. Highly intensive regimens tend to result in low diversity and richness [3,16].

Other studies indicate that the taxonomic distributions influence the risk of complications, but the results are inconclusive [3,11,16,17,18,19,20,21,22]. One reason as to why studies do not give consistent results can be the fact that the microbiome is functionally redundant, whereby several species can perform the same metabolic function [23]. For instance, many different bacterial species can produce acetate while firmicutes are the primary butyrate producers [24]. From a biological point of view, the function of the bacteria may be more important than the species itself [23,25]. If several bacterial species can perform the same function, and this function is important for the development of a disease, it will be difficult to detect this relationship in a study focusing solely on species distribution. Therefore, we believe that assessing the functional potential of the microbiome can be more informative than just the species distributions. We reckon metabolic functions to be most important as these are more likely to influence the immune system [26]. We refer to the estimated abundances of all metabolic functions in a sample as the metabolic potential profile. It shows which metabolic functions are present in the bacterial genomes in the sample, but it does not show if the genes are transcribed. Using whole genome shotgun sequencing, we can assess the metabolic potential by identifying the bacterial genes present in a stool sample and annotate the genes to metabolic functions.

In a previous study, we showed that patients who have undergone myeloablative conditioning had lower gene richness and lower abundance of Blautia after transplantation [3]. In that study, we only assessed the associations between conditioning regime and the bacteria we found to be associated with aGvHD risk. In the current study, we expanded the cohort with 114 patients and 172 samples and assessed the impact of the conditioning regimen on all species and diversity measures as well, as metabolic potential profiles. At our hospital, we use a low-dose truly non-myeloablative regimen and an intensive myeloablative regimen, allowing us to assess the impact of the intensity by stratification into myeloablative and non-myeloablative. Here, the aim was to provide new knowledge regarding associations of the intensity of the conditioning regimen and gut dysbiosis from a functional perspective.

## 2. Results

### 2.1. Patient Characteristics and Samples

A total of 278 patients delivered 678 stool samples in conjunction with their aHSCT between February 2016 and September 2020. After quality assessment of samples, 274 patients had 660 samples eligible for further analysis. Samples were split into two time periods, pre-aHSCT (day −30 to 0) and post-aHSCT (day 1 to 28). After dividing samples into these periods and filtering away those outside of the interval, there were 103 patients who underwent myeloablative conditioning and 151 patients who underwent non-myeloablative conditioning left. The myeloablative group was divided into 67 pre-aHSCT and 76 post-aHSCT samples. Out of the 103 patients undergoing myeloablative conditioning, 40 had both a pre-aHSCT and a post-aHSCT sample. There were 104 and 118 non-myeloablative pre-aHSCT and post-aHSCT samples, respectively. Out of the 151 patients who underwent non-myeloablative conditioning, 71 had both a pre-aHSCT and a post-aHSCT sample (Figure S1). The gender, underlying disease distributions and fraction of related donors were similar between the two conditioning regimens (Table 1). The mean age was significantly lower for the myeloablative group than for the non-myeloablative group (Table 1). For the post-aHSCT samples, the mean number of days of antibiotic prescriptions in the last 100 days prior to sampling was larger for the myeloablative than for the non-myeloablative group (Wilcoxon, *p* < 0.01, Figure S2).

### 2.2. Metabolic Potential Profiles

We use gut metabolic modules (GMMs) to annotate bacterial metabolic functions [27]. The most abundant GMM in our samples was lactose degradation (MF0007). Lactose degradation was present in all samples with a normalized abundance above 0.4 (Table 2). The next five most abundant metabolic functions were melibiose degradation, arabinoxylan degradation, mannose degradation, glycolysis, pyruvate:formate lyase and sucrose degradation I (Table 2). The potentially clinically relevant GMMs related to production of short chain fatty acids (SCFAs) and indole were present in many samples (Table 2). Indole is created by the degradation of tryptophan; we therefore use tryptophan degradation as a measure for indole production. In total, 92 different GMMs were present in at least 10% of the samples in our cohort (Figure S3).

### 2.3. Significant Differences in the Microbiome Diversity and Richness between Conditioning Regimens Were Only Observed Post-aHSCT

**Figure 1:**
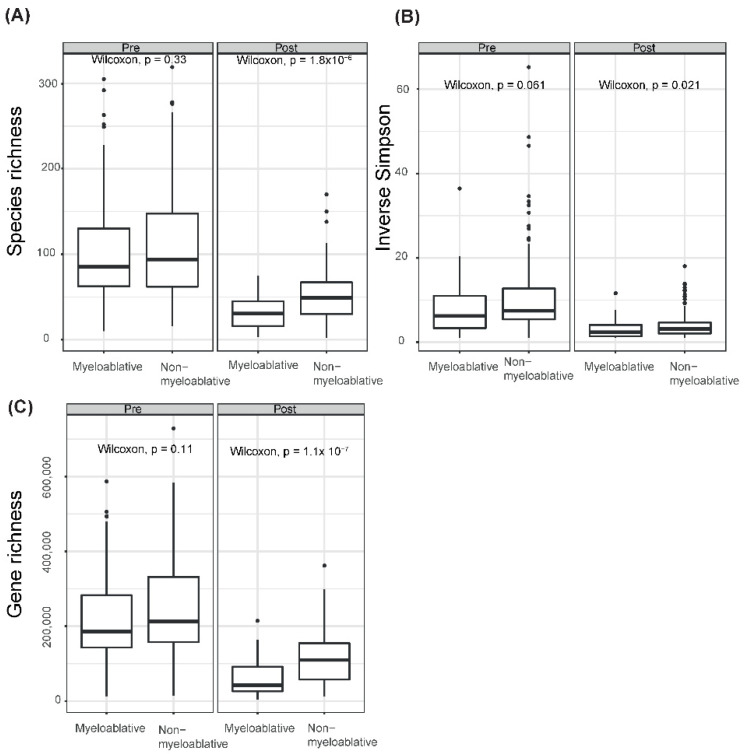
Gut microbiome diversity and associations with conditioning regimens. Boxplots of gut microbial diversity ((**A**) species richness, (**B**) alpha diversity and (**C**) gene richness) in patients undergoing myeloablative conditioning vs. non-myeloablative conditioning in the pre-aHSCT and post-aHSCT samples. Wilcoxon tests were performed per diversity measure and period.

For all samples, we estimated the species richness, alpha diversity and gene richness and compared them between conditioning regimen at each timepoint. In the pre-aHSCT samples, there were no significant differences between any of the measures (Figure 1). For the post-aHSCT samples, the means of all measures were significantly lower in the myeloablative group compared to the non-myeloablative group (Wilcoxon, *p* < 0.05, Figure 1).

### 2.4. The Taxonomical Distributions Were Similar between Conditioning Regimens before Transplantation but Differed Significantly after

**Figure 2:**
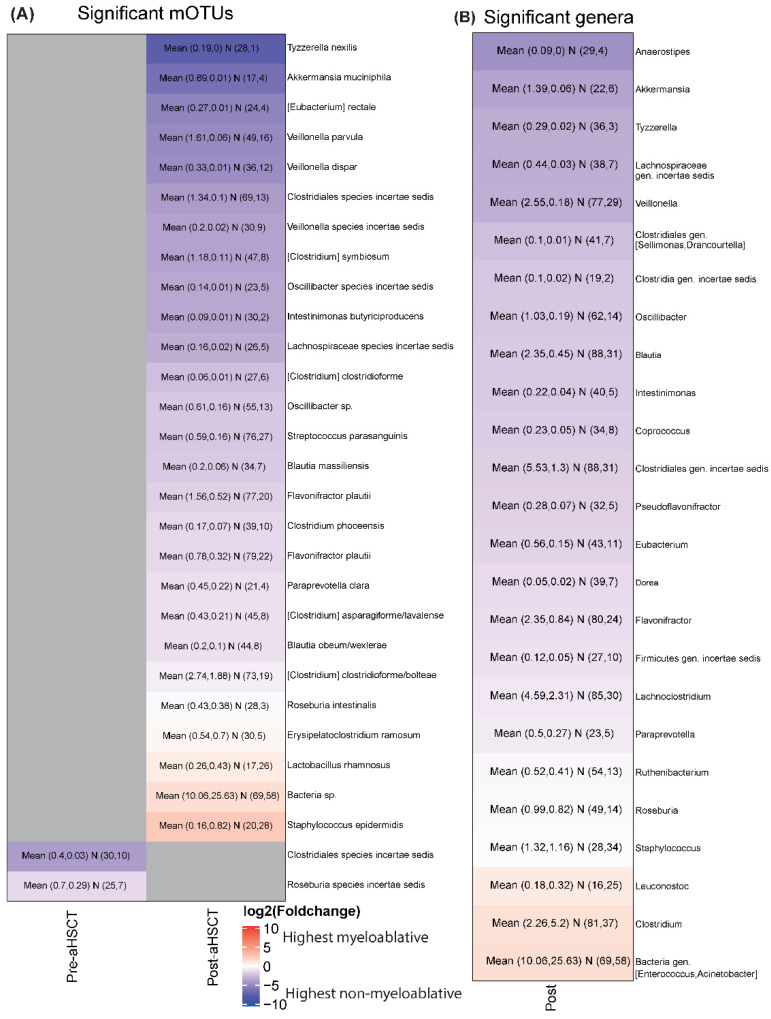
Taxonomic differences between conditioning regimens. (**A**) Heatmap on mOTU level of the log2 fold changes between the non-myeloablative and myeloablative groups in the pre-aHSCT and post-aHSCT sampling periods. The plot shows all mOTUs that are significantly different in at least one comparison using our discovery method. Gray means not significant. Blue colors show that the mean abundance of an mOTU is higher in the non-myeloablative group than in the myeloablative group and red shows the opposite direction. The text in the cells shows “Mean (mean (non-myeloablative), mean (myeloablative)) N (number of non-myeloablative samples with the mOTU present, number of myeloablative samples with the mOTU present)”. The total number of samples are pre-aHSCT myeloablative = 67, pre-aHSCT non-myeloablative = 104, post-aHSCT myeloablative = 76, post-aHSCT non-myeloablative = 116. (**B**) The same plot as A but on genus level. Only the post period is shown as no genera differed in the pre-aHSCT period.

We compared the taxonomical distributions on the genus and marker gene-based operational taxonomic unit (mOTU) level. In the pre-aHSCT period, the means of one Clostridiale species and one Roseburia species were significantly lower in the myeloablative group than in the non-myeloablative group (Figure 2A). For the post-aHSCT period, the means of 27 mOTUs and 25 genera were significantly different between the conditioning regimens (Figure 2A,B). Most mOTUs and genera were lower in the myeloablative group than in the non-myeloablative group (Figure 2A,B).

### 2.5. The Metabolic Potential Profiles and Metabolic Richness Were Similar between Conditioning Regimens before Transplantation but Differed Significantly after

**Figure 3:**
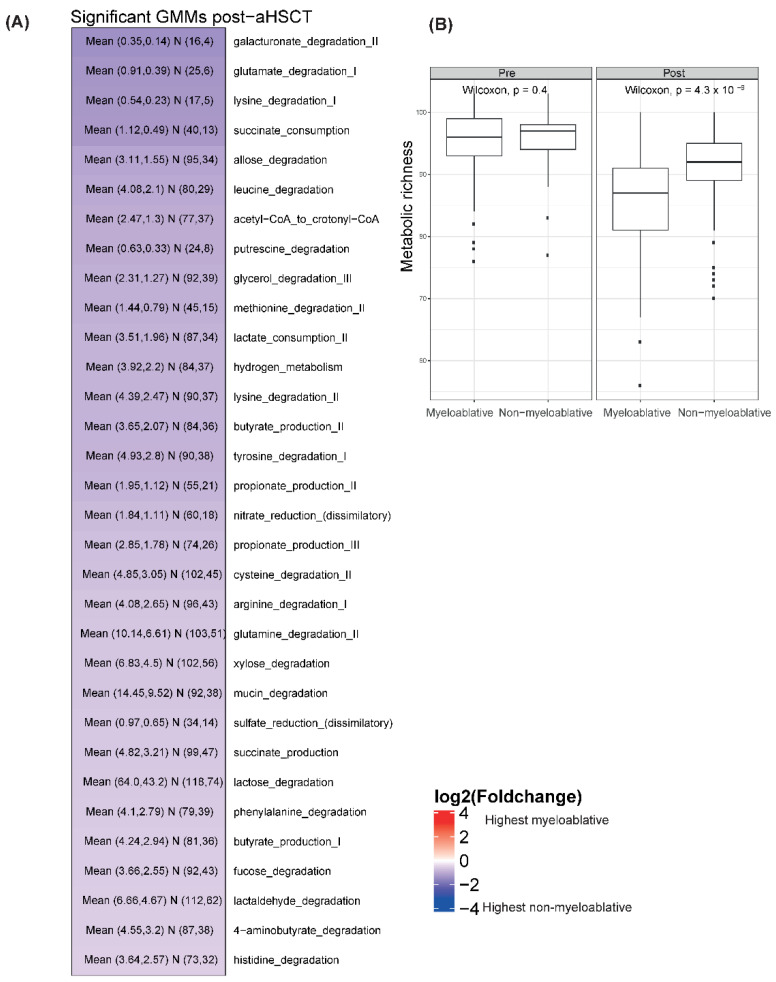
Associations between GMM profiles and conditioning regimens. (**A**) Heatmap on GMM level of the log2 fold changes between the non-myeloablative and myeloablative groups in the post-aHSCT sampling period, no comparisons were significant for the pre-aHSCT period. The plot shows all GMMs that are significantly different using a Wilcoxon test and have an absolute log2 fold change of above 0.5. Blue colors show that the mean abundance of a GMM is higher in the non-myeloablative group than in the myeloablative one and red shows the opposite direction. The text in the cells shows “Mean (mean (non-myeloablative), mean (myeloablative)) N (number of non-myeloablative samples with the GMM present, number of myeloablative samples with the GMM present)”. The total numbers of samples are pre-aHSCT myeloablative = 67, pre-aHSCT non-myeloablative = 104, post-aHSCT myeloablative = 76, post-aHSCT non-myeloablative = 116. (**B**) Boxplots of metabolic richness in patients undergoing myeloablative conditioning vs. non-myeloablative in pre-aHSCT and post-aHSCT samples. Wilcoxon tests were performed per time period.

In the pre-aHSCT samples, the allose degradation module was more abundant in the myeloablative group than in the non-myeloablative group (Wilcoxon, false discovery rate (FDR) = 0.006, log2 fold change of −0.24). For the post-aHSCT samples, 60 GMMs were significantly lower in the myeloablative group than in non-myeloablative group (Wilcoxon, FDR < 0.05, Figure 3A). The following four GMMs have a log2 (fold change) < −1: galacturonate degradation II, glutamate degradation I, lysine degradation I and succinate consumption. The metabolic richness (number of GMMs present) in the post-aHSCT samples was higher in the non-myeloablative group than in the myeloablative group (Wilcoxon, *p* < 0.0001, Figure 3B).

### 2.6. Myeloablative Conditioning Was Associated with Larger Differences in Richness and Diversity Measures than Non-Myeloablative Conditioning

**Figure 4:**
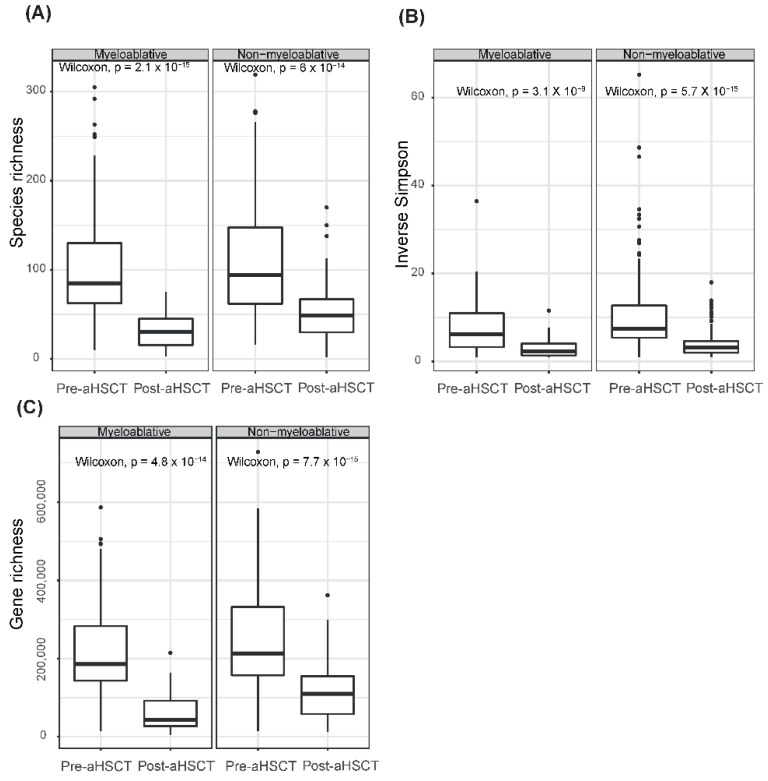
Gut microbiome diversity and associations with timepoints. Boxplots of gut microbial diversity ((**A**) species richness, (**B**) alpha diversity and (**C**) gene richness) in pre-aHSCT vs. post-aHSCT samples for the myeloablative and non-myeloablative groups. Wilcoxon tests were performed per diversity measure and conditioning regimen.

We showed that the microbiome differed between the conditioning regimens after the transplantation but not before. Next, we wanted to explore how the two conditioning regimens were associated with differences in the microbiome over time. We compared the species richness, alpha diversity and gene richness for each regimen between the pre-aHSCT and post-aHSCT samples. The diversity measures for both regimens declined over time (Wilcoxon, *p* < 0.0001, Figure 4). For the myeloablative group, the species richness was 3.2 times higher pre-aHSCT than post-aHSCT and for the non-myeloablative group the richness was 2.2 times higher pre-aHSCT than post-aHSCT. The inverse Simpson index was 2.6 times higher pre-aHSCT than post-aHSCT for both groups. For the myeloablative group, the gene richness was 3.5 times higher pre-aHSCT than post-aHSCT and for the non-myeloablative group the richness was only 2.1 times higher pre-aHSCT than post-aHSCT. To evaluate responses in individual patients, we conducted a paired analysis using only patients that had both a pre-aHSCT and post-aHSCT sample. The paired samples also showed decline in all measures for all groups between pre-aHSCT and post-aHSCT (paired Wilcoxon, *p* < 0.001, Figure S4A–C).

### 2.7. Myeloablative Conditioning Was Associated with Larger Changes in Taxonomical Distributions than Non-Myeloablative

**Figure 5:**
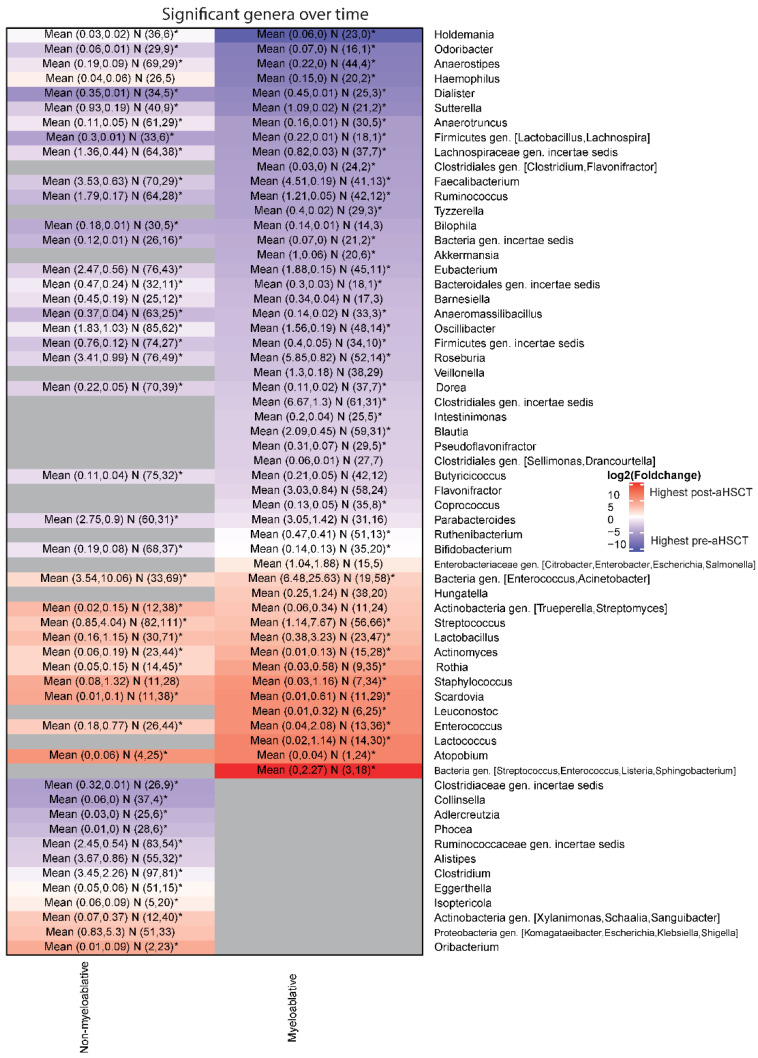
Associations of taxonomy with timepoints. Heatmap on genus level of the log2 fold changes between pre-aHSCT and post-aHSCT sampling period stratified by conditioning regimen. The plot shows all genera that are significantly different in at least one comparison using our discovery method. Gray means not significant. Blue colors show that the mean abundance of a genus is higher pre-aHSCT than post-aHSCT and red shows the opposite direction. The text in the cells shows “Mean (mean (pre-aHSCT), mean (post-aHSCT)) N (number of pre-aHSCT samples with the genera present, number of post-aHSCT samples with the genera present). A star indicates that the comparison is also significant in the paired analysis”. The total numbers of samples were pre-aHSCT-myeloablative = 67, pre-aHSCT-non-myeloablative = 104, post-aHSCT-myeloablative = 76, post-aHSCT-non-myeloablative = 116.

For the myeloablative group, 36 genera declined while 15 genera increased over time with a mean absolute log2 fold change of 3.9. For the non-myeloablative group, 30 genera declined while 16 genera increased over time with a mean absolute log2 fold change of 2.4 (Figure 5). Of the 63 genera, 34 were common between the regimens, and except for Haemophilus, the fold changes had the same direction in all cases. The relative abundance of Haemophilus decreased in the myeloablative group (log2 fold change = −6.8) while it had a modest increase for the non-myeloablative group (log2 fold change = 0.6). The paired analysis was significant for 41 and 43 of the significant genera for the myeloablative and the non-myeloablative groups, respectively (Figures S5 and S6). In general, genera with high fold changes were more likely to be significant in the paired analysis. On the mOTU level, 93 mOTUs decreased and 20 increased in the myeloablative group (Table S1). The mean absolute log2 fold change was 4.3. For the non-myeloablative group, 73 mOTUs decreased, 21 increased and the mean absolute log2 fold change was 2.8 (Table S2). The paired analysis was significant for 71 and 83 of the mOTUs for the myeloablative and non-myeloablative groups, respectively (Tables S1 and S2).

### 2.8. Myeloablative Conditioning Was Associated with Larger Changes in Metabolic Potential Profiles and Metabolic Richness Than the Non-Myeloablative Regimen

**Figure 6:**
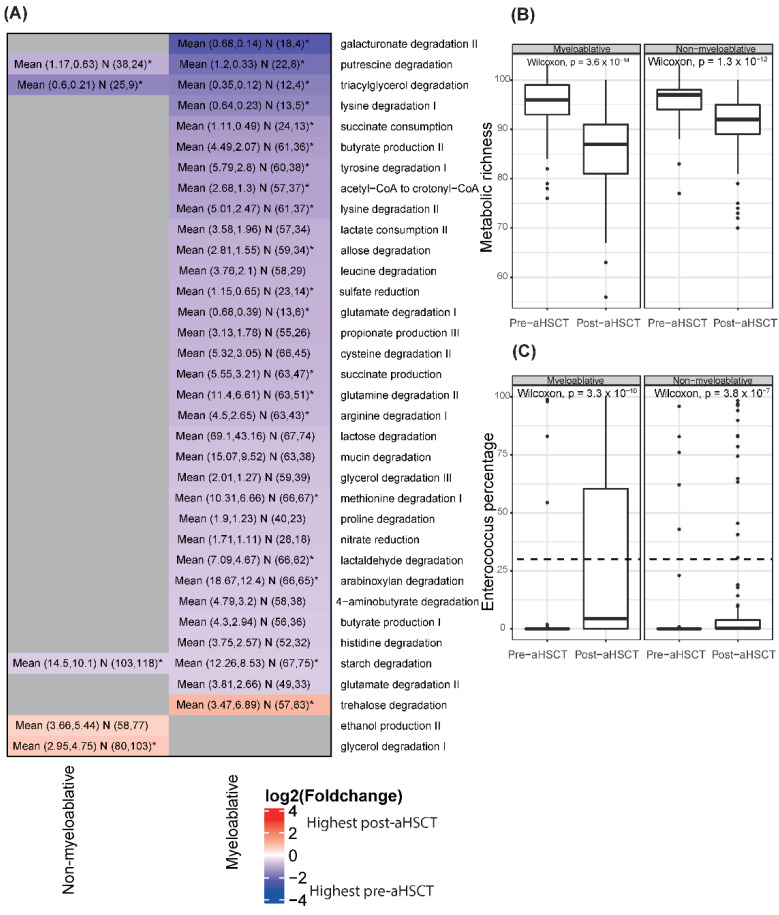
Associations of GMMs and Enterococcus profiles with timepoints**.** (**A**) Heatmap on GMM level of the log2 fold changes between the pre-aHSCT and post-aHSCT sampling period stratified by conditioning regimen. The plot shows all GMMs that are significantly different in at least one comparison based on a Wilcoxon test. Gray means not significant. Blue colors show that the mean abundance of a GMM is higher in the pre-aHSCT period than in the post-aHSCT period and red shows the opposite direction. The text in the cells shows “Mean (mean (pre-aHSCT), mean (post-aHSCT)) N (number of pre samples with the GMM present, number of post samples with the GMM present). A star indicates that the comparison is also significant in the paired analysis”. The total numbers of samples are pre-aHSCT myeloablative = 67, pre-aHSCT non-myeloablative = 104, post-aHSCT myeloablative = 76, post-aHSCT non-myeloablative = 116. (**B**) Boxplots of metabolic richness in pre-aHSCT vs. post-aHSCT samples for myeloablative patients and non-myeloablative patients. Wilcoxon tests were performed per conditioning regimen. (**C**) Boxplots of the percentage of Enterococcus in pre-aHSCT vs. post-aHSCT samples for patients undergoing myeloablative and non-myeloablative conditioning. Wilcoxon tests were performed per conditioning regimen. The dotted lines indicate the threshold for domination by Enterococcus (30%).

For the myeloablative group, 32 GMMs declined while one GMM increased over time with a mean absolute log2 fold change of 0.9 (Figure 6A). Out of the 33 GMMs, 20 were significant in the paired analysis (Figure S7). For the non-myeloablative group, three GMMs declined while two GMMs increased over time with a mean absolute log2 fold change of 0.8 (Figure 6A). Four out of the five GMMs were significant in the paired analysis (Figure S8). Only three GMMs were common between the regimens and they all declined. The metabolic richness declined over time for both regimens (Figure 6B and Figure S4D).

### 2.9. The Genus Enterococcus Is Present in a High Number of Patients Pre-aHSCT and Becomes Dominant in Many Patients Post-aHSCT

As enterococcal dominance has been associated with a worse outcome in this patient group, we chose to investigate this genus. It is normal for Enterococcus to make up a small proportion of the gut microbiome (<0.1) [15]. We detect Enterococcus in amounts higher than 0.1% in 40% of the myeloablative pre-aHSCT samples and in 41% of the non-myeloablative pre-aHSCT samples. For the post-aHSCT samples, the numbers are 84% and 68% for the myeloablative and non-myeloablative groups, respectively. In some studies, it was found that Enterococcus becomes the dominating species (>30%) in some patients after aHSCT [3,15]. For our study, the percentage of post-aHSCT samples with Enterococcus as the dominating genus was 38% and 14% for the myeloablative and non-myeloablative groups, respectively (Figure 6C). For the pre-aHSCT samples, the numbers are 7% and 5% for the myeloablative and non-myeloablative groups, respectively (Figure 6C). The mean percentage of Enterococcus is significantly higher post-aHSCT than pre-aHSCT for both regimens (Wilcoxon, *p* < 0.0001, Figure 5 and Figure 6C).

### 2.10. Associations between Enterococcus, Lactose Degradation and Conditioning Regimen

We found lactose degradation to be the most prominent metabolic function. Lactose is known to be an important substrate for Enterococcus and it has been shown that lactose drives Enterococcus expansion [15]. We found the abundance of the GMM lactose degradation (MF0006) was negatively correlated with the percentage of Enterococcus in the sample (Spearman rho = −0.48, *p* < 0.0001, Figure S9A). The GMM lactose and galactose degradation (MF0007), on the other hand, was positively correlated with the percentage of Enterococcus in the sample (Spearman rho = 0.48, *p* < 0.0001, Figure S9B). For the myeloablative group, the normalized abundance of MF0006 was lower post-aHSCT than pre-aHSCT (Wilcoxon, *p* < 0.0001, Figure S10).

## 3. Discussion

In this study, we applied a metagenomic approach to access gut microbial taxonomy, diversity and metabolic potential profiles in a large aHSCT cohort. We found substantial differences in all measures post-aHSCT, but not pre-aHSCT, between patients undergoing a non-myeloablative and a myeloablative conditioning regimen. Therefore, we explored the changes over time individually for the two regimens. We found the myeloablative regimen was associated with more taxonomic changes than the non-myeloablative regimen and the mean fold changes were also larger for the myeloablative group. On the functional level, the difference between groups was even more pronounced. Around six times more metabolic functions changed over time for the myeloablative group compared to the non-myeloablative group. Previous studies have shown that the species diversity and the species and gene richness of fecal microbiomes decline during stem cell transplantation [3,10,28]. Previously, we have also shown that a myeloablative regimen is associated with a more pronounced decline in gene richness than a less intense non-myeloablative regimen [3]. Furthermore, we saw a similar trend for the metabolic richness [3]. In the current extended cohort, we validated the associations with gene and metabolic richness as well as demonstrated novel associations with species richness and alpha diversity.

We observed clear changes in the taxonomical distributions over time, indicating that the same species increased or decreased during the transplantation course for larger groups of patients. One clinically important change was the relative decrease in the genus Blautia in patients undergoing myeloablative conditioning. Blautia has been associated with risk of developing aGvHD [3,21,29]. At the species level, three Blautia species decreased (Blautia obeum/wexlerae, Blautia producta and Blautia massiliensis), but only Blautia obeum/wexlerae was also significant in the paired analysis.

Another important observation was that the genus Enterococcus was present in higher amounts than normally found in healthy microbiomes in a large proportion of samples. For the myeloablative post-aHSCT group, as many as 84% had Enterococcus present in a higher amount than normal. However, the percentages of samples with Enterococcal domination post-aHSCT were in the same range as seen in previous studies [3,15]. As lactose has been shown to drive Enterococcus expansion [15], we explored associations between lactose degradation and Enterococcus. There are two pathways through which bacteria can degrade lactose, the lactose degradation pathway (GMM MF0006) and the lactose and galactose degradation pathway (GMM MF0007). MF0006 is the GMM with the highest abundance across our samples and it has a negative correlation with Enterococcus. According to metaCyc, Enterococcus and other Firmicutes use MF0007 instead of MF0006 [30]. Therefore, it is not unexpected that MF0006 is not positively correlated with Enterococcus. As expected, MF0007 is positively correlated with the abundance of Enterococcus. The high abundance of MF0006 shows that other bacteria capable of degrading lactose are present, and it indicates that there is a high amount of lactose in the intestines of the patients from our cohort. Danes generally consume products rich in dairy, explaining the concentration of lactose consumers. The high amount of lactose is probably the reason for the unexpected generally high percentage of Enterococcus in this cohort. It has been hypothesized that more lactose than normal reaches the lower intestinal tract post-aHSCT as the small-intestinal enterocytes that produce lactase are damaged by the conditioning [15]. This is thought to be part of the explanation why Enterococcus dominates post-aHSCT.

The changes in taxonomic distributions were reflected in the metabolic potential profiles, but the effect was not as pronounced. More than 30 metabolic functions changed for the myeloablative regimen, but the absolute fold changes were low and when looking at the paired samples the patterns are not as clear as for the taxonomy. However, we observed a decrease in metabolic richness, revealing that some functions disappeared in individual samples, but that they are not the same functions for larger groups of patients. As for the other measures, the differences in metabolic potential profiles were less pronounced in the non-myeloablative group. Only five GMMs changed over time for the non-myeloablative group.

Both the overall disturbances of the metabolic profiles and the changes in individual functions could be important in relation to clinical outcomes. Indole derivatives and SCFAs are thought to be the most important metabolites produced by gut bacteria that are known to influence gut health [24,26,29]. Hence, changes in SCFA and indole production could have clinical impact. Indole derivatives have been shown to be important for the prevention of aGvHD [29]. We detect the potential for indole production in low amounts in most samples, but we do not see a decrease over time or a difference between the regimens in indole production. This indicates that both conditioning groups could benefit from treatments that increase indole levels.

We detected the potential for production of the SCFAs acetate, propionate and butyrate in many samples, but we only found differences over time for butyrate formation. Butyrate formation has been shown to be associated with an increase in white blood cells [2]. Another study found that diversity was associated with butyrate levels and low diversity at engraftment increased the risk of aGvHD [8]. Other studies, both in mice and humans, also indicate the important role of butyrate in mitigating and preventing aGvHD [31]. We found the GMMs butyrate production I and II decreased in patients undergoing myeloablative conditioning, but not in the non-myeloablative group. Post-aHSCT, the mean normalized values for butyrate production I and II were also lower for the myeloablative than for the non-myeloablative group. These are novel observations and could be part of the explanation for why the aGvHD risk is high for the myeloablative group. The study by Yoshifuji et al. indicates that some prebiotics can increase butyrate levels and mitigate aGvHD [32]. However, their study included a relatively small cohort (N = 32 for the prebiotic group and N = 72 for the control group). Further, they use a historical control cohort and the change in treatment and eating habits over time is not accounted for. Future studies and randomized clinical trials with larger cohorts are needed to investigate if treatments that increase the butyrate level mitigate aGvHD. Our results indicate that such treatments are most likely to have an effect in the myeloablative group, and we would recommend to primarily include patients undergoing myeloablative conditioning in such studies or stratify by conditioning regimen.

There can be several reasons why the myeloablative regimen has a larger impact on the microbiome than the non-myeloablative regimen. The regimens differ in radiation, chemotherapy, medication usage, age distribution, length of hospitalization and number of patients in need of total parental nutrition. All these factors could potentially influence the microbiome. Given the tight associations between the regimen and these factors, we are not able to untangle the effect of the different factors. We can only conclude that the microbiome of the patients who undergo myeloablative conditioning changes more than the microbiome of the patients who undergo non-myeloablative conditioning. However, one important difference is that the myeloablative group received more antibiotics around and just after the time of transplantation. This is partly because they were treated with ceftazidime during the neutropenic state and in addition they had a higher risk of severe infections as they experienced more intense immune suppression. We believe the heavy use of antibiotics in the myeloablative group is the most likely reason for the differences in the microbiomes between the groups as antibiotics are known to cause gut dysbiosis [33]. Time of year and time of day have also been shown to influence the microbiome [34,35] but we believe the impact to be negligible compared to the clinical factors and samples are randomly distributed over the year.

We are studying the function of the microbiome through the prediction of metabolic potential profiles. The profiles only show what the microbiome has the potential to do. They do not tell if the genes are functional and if they are actively expressed under the current conditions. More direct approaches to study the function of the microbiome would require metatranscriptomics, metaproteomics or metabolomics [25]. However, these methods are costly and not fully matured [25], therefore we use metabolic potential profiles to get a step closer to studying the function of the microbiome without the need to perform more costly omics experiments. Another limitation of our study is the small number of consecutive samples per patient.

Here, we apply metabolic potential analysis to collapse numerous bacterial species of varying relation into categories of metabolic functions, enabling assessment of ~110 different metabolic functions. In terms of functionality, bacteria show high redundancy [23]. Hence, we hypothesized that this approach would give us a better biological understanding of the processes that are involved in gut dysbiosis in this patient group than the conventional taxonomic approach. Our study validates some of the changes seen by others in the taxonomical features during the transplant course as well as showing that the taxonomical changes are reflected in changes in metabolic potential profiles. The intensity of the conditioning regimen is associated with the degree of these changes and suggests that, in terms of a functionally healthy microbiota, some optimal intensity could be found.

## 4. Materials and Methods

### 4.1. Inclusion of Patients

Adults (≥18 years) who underwent a first aHSCT at the Stem Cell Transplantation Unit, Department of Haematology, Copenhagen University Hospital, Rigshospitalet, Denmark between 1 February 2016 and 1 September 2020 were prospectively included.

### 4.2. Transplant Procedures

All patients underwent either myeloablative or non-myeloablative conditioning prior to transplantation. The main myeloablative conditioning regimens were fludarabine combined with treosulfan, cyclophosphamide with total body radiation (TBI) (1200 Gray (Gy)) or cyclophosphamide with TBI (1200 Gy) and anti-thymocyte globulin (ATG). The main non-myeloablative conditioning regimen was TBI (≤400 Gy) and fludarabine. Gut decontamination was not performed prior to aHSCT. Patients received antibiotics when neutrophil counts were <0.5 × 10^9^/L post-aHSCT (myeloablative conditioning: ceftazidime 2 g IV3 times daily, non-myeloablative: ciprofloxacin 500 mg × 2 p.o. daily) until neutrophil counts were >0.5 × 10^9^/L. All patients received sulfamethoxazole and trimethoprim 400/80 mg × 1 p.o. daily from day 0 until the end of immunosuppressive treatment.

### 4.3. Antibiotic Usage

Prescriptions of antibiotic usage were extracted from electronic health care records. Prescriptions were grouped into (1) broad spectrum beta lactam, given intravenously and (2) other antibiotics. For each sample, the numbers of prescription days for each group for the previous 100 days were counted.

### 4.4. Sample Collection and Sequencing

Fecal samples were collected by the patient or nursing staff using the OMNIgene.GUT (DNA Stabilized-frozen Inc., Ottawa, ON, Canada) stabilization tube according to the manufacturer’s instructions and refrigerated for a maximum of 7 days before freezing at −80 °C. Of 660 samples with enough reads, 29 had a missing sampling date and received the freezing date (i.e., the date the sample was frozen) as a proxy sample date. Samples were stored at −80 °C until shipment for sequencing. Samples underwent paired end sequencing on an Illumina Hi-Seq^®^ with a read length of 150 bp.

### 4.5. Pre-Processing and Quality Control

Reads were pre-processed and analyzed using our in-house microbiome profiling pipeline implemented in NGLess and inspired by NG-meta-profiler [36]. First reads were cut at positions where the average quality score over four bases was less than 20. Then, the longest part of each read was trimmed from the ends using a quality score cut-off of 20. Reads below 100 bases were discarded. Reads mapping to the human genome (hg19) using the BWA-MEM2 alignment tool [37] and with a minimum identity of 80 across 90 bases were also discarded.

### 4.6. Taxonomy, Richness and Diversity Measures

Taxonomical profiles on the genus and mOTU level were assigned using mOTUs2 [38]. The naming for mOTUs gives all possible species that the specific mOTU potentially represents. The format is species1/species2/species3/etc., meaning that all the species are possible annotations. To ease the interpretation, we have modified the format to species1, species2, species3 in the main text and figures. When we counted the number of samples having Enterococcus present, we considered all mOTUs that were annotated as potentially Enterococcus. Diversity (inverse Simpson) and richness (observed number of species) were calculated on the mOTU level using Phyloseq [39]. Gene profiling was carried out by mapping to the integrated gene catalogue (IGC) [40] with BWA-MEM2 [37] and counting the number of reads mapping to each gene with a minimum match size of 60 and minimum identity of 90. Gene richness was calculated by sampling 4,000,000 gene names from the gene profiles and counting the number of unique genes.

### 4.7. Metabolic Potential Profiles and Metabolic Richness

We used gut metabolic modules (GMMs) to profile the metabolic potential [27]. GMM/metabolic profiling was carried out by length normalizing the IGC count profiles and summing the values for each KEGG [41] gene ontology term. Afterwards, the values were 16sRNA normalized and turned into GMM profiles using omixer-RPM [27]. To find the 16sRNA normalization factor for each sample, the reads were first mapped with KMA [42] to the Silva bacteria SSU database (downloaded on the 9 November 2020) [43]. Subsequently, 16sRNA genes from the same genomes were combined into one gene, followed by length normalization of the reads. Finally, values were summed and multiplied by 100. The normalization did not consider that some genes, including the 16sRNA gene, can be present many times in the same genome. Hence, the normalized values are not strictly percentages, but they are to our knowledge the best possible available proxy for the percentage of bacteria in a sample that can perform a specific function. Metabolic richness was calculated using the observed function from Phyloseq on the metabolic profiles [39].

### 4.8. Statistical Analysis

Patients were included if they had at least one sample, collected within day −30 to +28 that passed quality assessment. Samples were grouped into the following time periods: pre-aHSCT (day −30 until and including day 0) and early post-aHSCT (day 1 until and including day +28). One sample per patient per period was selected. For patients with multiple samples per period, the median time of sampling in patients with one sample was calculated and the sample closest to this median time (−17 days for pre-aHSCT and 16 days for post-aHSCT) was selected.

Unless otherwise stated, all statistical tests were corrected for multi-testing using Benjamini–Hochberg FDR corrections [44] and a significance threshold of 0.05.

We tested for differenced in taxonomy on the genus and mOTU level using a combination of two methods, similar to the discovery method used in our previous study [3]. The method required a taxonomical unit to be significantly different both using DESeq2 [45] and a Wilcoxon test, taking compositionality into account [46], with a combined significance cut-off of FDR < 0.1 and *p* ≤ 0.05, respectively. The input for DESeq was raw counts and the input for the Wilcoxon test was relative abundances. Taxonomical units were compared between patient groups and timepoints. For each comparison, genera and mOTUs that were present in a minimum of 10% of the samples were included in the analysis. We define presence as an abundance of 0.01% for the Wilcoxon test and >0 reads for the DESeq2 test. The significant genera were visualized with paired boxplots using only the patients with both a pre-aHSCT and a post-aHSCT sample. The abundances were compared between timepoints using a paired Wilcoxon test.

All four diversity and richness measures (species richness, gene richness, species diversity and metabolic richness) were compared between patient groups and timepoints using a Wilcoxon test with no correction for multiple testing (α = 0.05).

The GMM abundances were compared between patient groups and timepoints using a Wilcoxon test. GMMs with a normalized abundance>1 in a minimum of 10% of the samples were included in the analysis. An absolute log2 fold change of above 0.5 was required for a GMM to be significant. The significant GMMs were compared with a paired Wilcoxon test and visualized with paired boxplots using only the patients with both a pre-aHSCT and a post-aHSCT sample.

## Figures and Tables

**Table 1:** Patient characteristics.

	Myeloablative (N = 103)	Non-Myeloablative (N = 151)	Total (N = 254)	*p*-Value
Age in years				<0.001
Mean (SD)	49.2 (13.18)	61.8 (10.25)	56.7 (13.06)	
Min–max	21–71	23–78	21–78	
Gender				0.238
Female	50 (48.5%)	62 (41.1%)	112 (44.1%)	
Male	53 (51.5%)	89 (58.9%)	142 (55.9%)	
Disease				0.778
Acute leukemia	40 (38.8%)	56 (37.1%)	96 (37.8%)	
Other	63 (61.2%)	95 (62.9%)	158 (62.2%)	
Donor relationship				0.745
Matched related donor	25 (24.3%)	34 (22.5%)	59 (23.2%)	
Matched unrelated donor	78 (75.7%)	117 (77.5%)	195 (76.8%)	

**Table 2:** Quantiles for the six most abundant and six a priori interesting GMMs.

	GMM	Detected (>0, >1) in N Samples	Min.	1st Qu.	Median	Mean	3rd Qu.	Max.
	Lactose Degradation	365,362	0.47	13.76	56.74	61.74	97.57	292.9
	Melibiose Degradation	365,351	0.01	8.2	16.43	16.61	22.28	84.68
The six most abundant GMMs	Arabinoxylan Degradation	365,341	0	6.38	15.87	16.4	24.77	67.19
	Mannose Degradation	365,361	0.28	6.97	15.42	15.43	21.52	48.54
	Glycolysis (Preparatory Phase)	365,363	0.51	11.37	15.23	15.57	18.95	53.05
	Pyruvate:Formate Lyase	365,363	0.4	10.36	13.06	13.38	16.29	42.74
	Propionate Production I	115,14	0	0	0	0.12	0.01	4.34
	Propionate Production II	334,145	0	0.19	0.71	1.57	1.84	18.94
Six a priori GMMs	Butyrate Production I	356,270	0	0.86	4.54	4.14	6.53	14.91
	Butyrate Production II	326,280	0	1.51	4.26	3.81	5.41	27.05
	Acetyl-Coa To Acetate	365,361	0.35	5.9	8.91	8.49	10.84	22.5
	Tryptophan Degradation	339,201	0	0.09	1.52	2.23	3.55	12.36

## Data Availability

The datasets generated and analyzed during this study are derived from patients treated in Denmark. The datasets contain sensitive patient data governed by GDPR and Danish law. Due to Danish legislation (Act No. 502 of 23 May 2018) and approvals granted by the Danish Data Protection Agency, it is not possible to upload raw data to a publicly available database. However, access to these data can be made available from the corresponding author on reasonable request, provided a data transfer agreement is entered into according to current regulations.

## Supplementary Materials

The supporting information can be downloaded at: https://www.mdpi.com/article/10.3390/ijms231911115/s1.
