# Supplementary material for: Metabolic Potential of the Gut Microbiome Is Significantly Impacted by Conditioning Regimen in Allogeneic Hematopoietic Stem Cell Transplantation Recipients
Source: Int J Mol Sci. 2022 Sep 21;23(19):11115. doi: 10.3390/ijms231911115 (PMC9570131; doi:10.3390/ijms231911115)
Supplement: Supplementary file 1 [file ijms-23-11115-s001.zip › Supplementary_figure_legends.pdf]

Supplementary figure legends

Supplemental Figure S1. Consort chart of patients and samples

The first row shows all patients and samples from aHSCT recipients transplanted at Rigshospitalet from Feb 2016 to Aug 2020. The first step filters away samples with less than 1 M reads. In the next step one sample per patient per time period is chosen. In the last step the samples are divided into samples from myeloablative and non-myeloablative patients. The number of new samples shows the number of samples that was not part of our previous cohort[10]. The number of pairs show the number of patients from each group, who has both a pre-aHSCT and post-aHSCT sample.

Supplemental Figure S2. Boxplots of antibiotic usage divided by conditioning regime and timepoint

Boxplots of days of antibiotic treatment in the last 100 days before the sample is taken divided by timepoint and conditioning. Panel A shows days of IV beta lactam treatment while B shows days of other antibiotic treatments.

Supplemental Figure S3. Boxplot of normalized GMM abundance

Boxplot of the log 2 (normalized abundance+1) of the 92 GMMs present in at least 10 percent of all samples with a normalized abundance of 1. The boxplot it ordered after the medians with the most abundant function at the top.

Supplemental Figure S4. Paired boxplots of all diversity measures divided by conditioning regimen and timepoint

Paired boxplots of diversity measures (A species richness, B species diversity, C Gene richness per 4M reads, D Metabolic richness) divided by conditioning regimen and timepoint. The plots only show samples from patients that had both a pre-aHSCT and a post-aHSCT sample

Supplemental Figure S5. Paired boxplots of the all genera that differed significantly between pre-aHSCt and post-aHSCT samples for the myeloablative patients

Paired boxplots for the 49 genera that differed significantly with an absolute log2 foldchange>0.5 between pre-aHSCt and post-aHSCT samples for the myeloablative patients. Of the 49 genera 41 were validated with a paired Wilcoxon test (FDR<0.05)

Supplemental Figure S6. Paired boxplots of the all genera that differed significantly between pre-aHSCt and post-aHSCT samples for the non-myeloablative patients

Paired boxplots for the 44 genera that differed significantly with an absolute log2 foldchange>0.5 between pre-aHSCt and post-aHSCT samples for the non-myeloablative patients. Of the 44 genera 43 were validated with a paired Wilcoxon test (FDR<0.05).

Supplemental Figure S7 Paired boxplots of the GMMs that differed significantly between pre-aHSCT and post-aHSCT for the myeloablative patients

Paired boxplots for the 33 GMMs that differed significantly with an absolute log2 foldchange>0.5 between pre-aHSCt and post-aHSCT samples for the myeloablative patients. Of the 33 GMMs 20 were validated with a paired Wilcoxon test (FDR<0.05).

Supplemental Figure S8 Paired boxplots of the GMMs that differed significantly between pre-aHSCT and post-aHSCT for the non-myeloablative patients

Paired boxplots for the 5 GMMs that differed significantly with an absolute log2 foldchange>0.5 between pre-aHSCT and post-aHSCT samples for the non-myeloablative patients. Of the 5 GMMs 4 were validated with a paired Wilcoxon test (FDR<0.05).

Supplemental Figure S9 Associations between lactose degradation and percentage of Enterococcus

Associations between the two detected lactose degradation pathways and the percentage of Enterococcus in the samples. Panel A shows MF0006 and panel B MF0007.

Supplemental Figure S10 Boxplots of the two lactose degradation pathways divided by timepoint and conditioning regimen

Boxplots of the normalized abundance of the two lactose degradation pathways divided by timepoint and conditioning regimen. Wilcoxon tests are done for differences in mean between timepoints for each combination of pathway and conditioning regimen.
