# Supplementary figures and images for: Metabolic Potential of the Gut Microbiome Is Significantly Impacted by Conditioning Regimen in Allogeneic Hematopoietic Stem Cell Transplantation Recipients
Source: Int J Mol Sci. 2022 Sep 21;23(19):11115. doi: 10.3390/ijms231911115 (PMC9570131; doi:10.3390/ijms231911115)

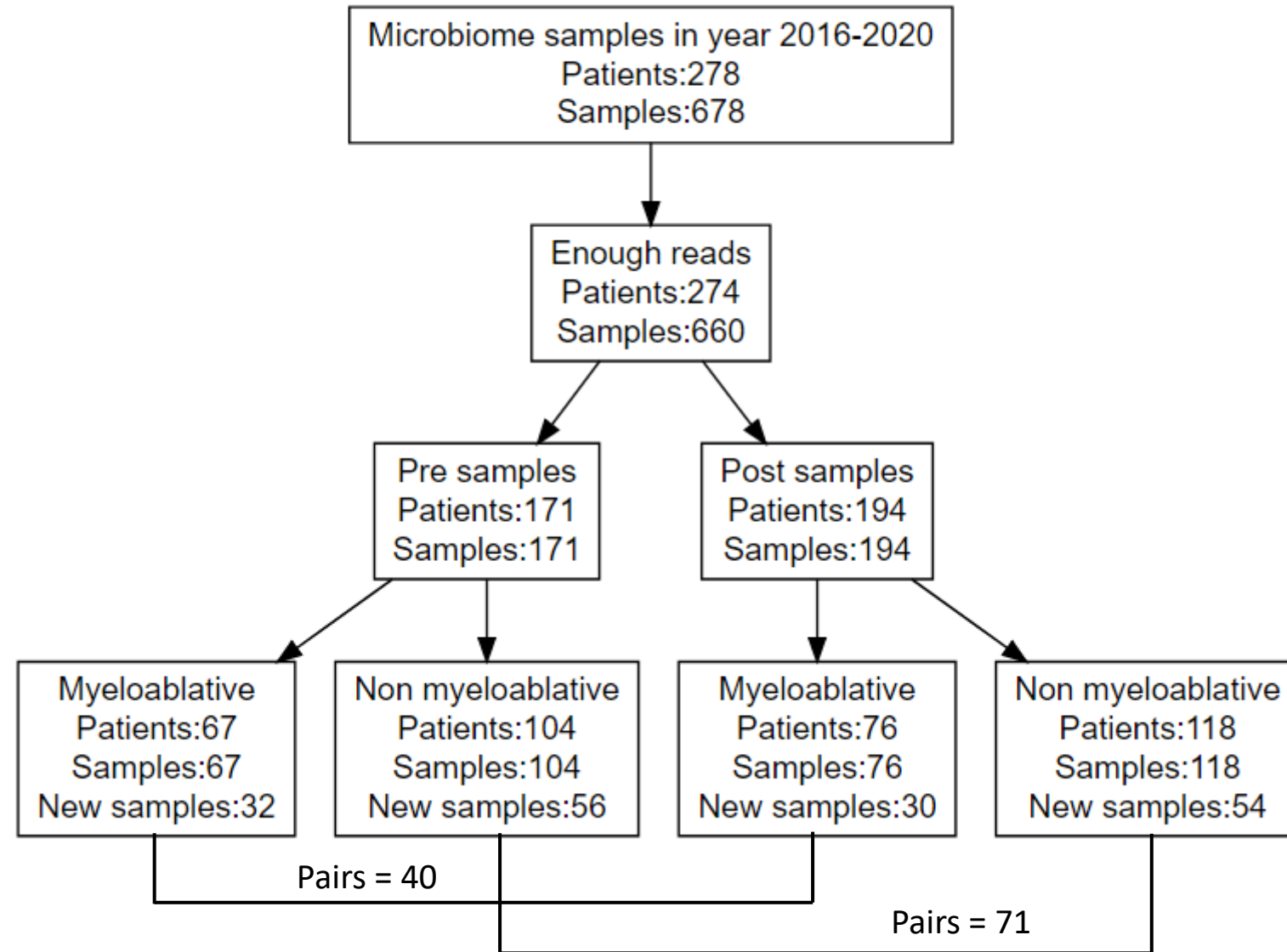

Figure S1

Supplement: Supplementary file 1 [file ijms-23-11115-s001.zip › S1.pdf]

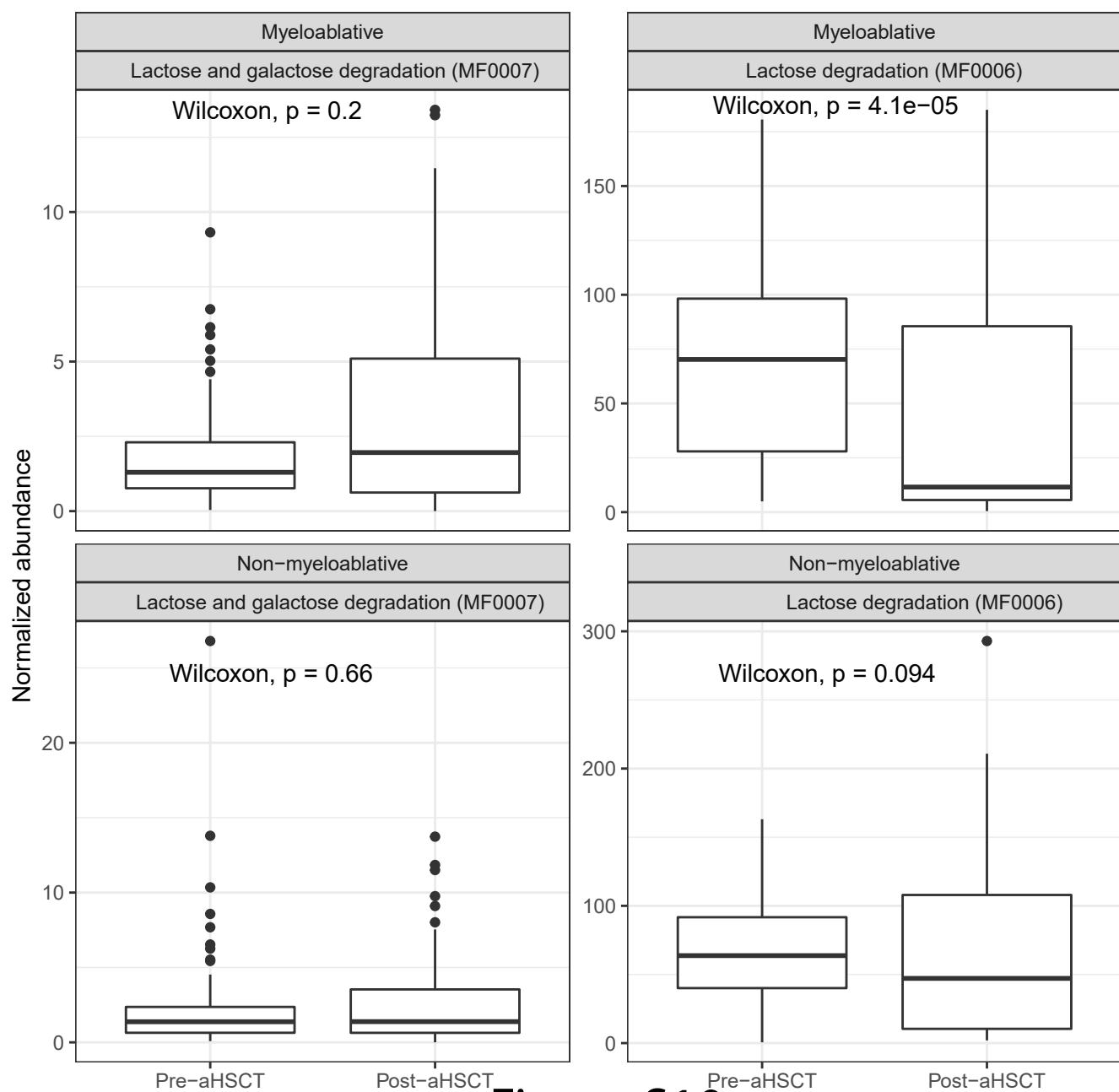

Figure S10

Supplement: Supplementary file 1 [file ijms-23-11115-s001.zip › S10.pdf]

A)

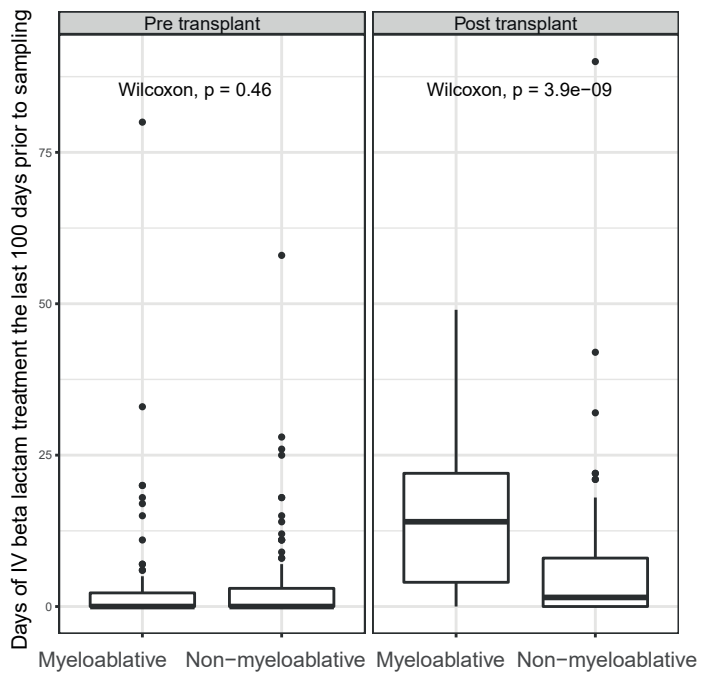

B)

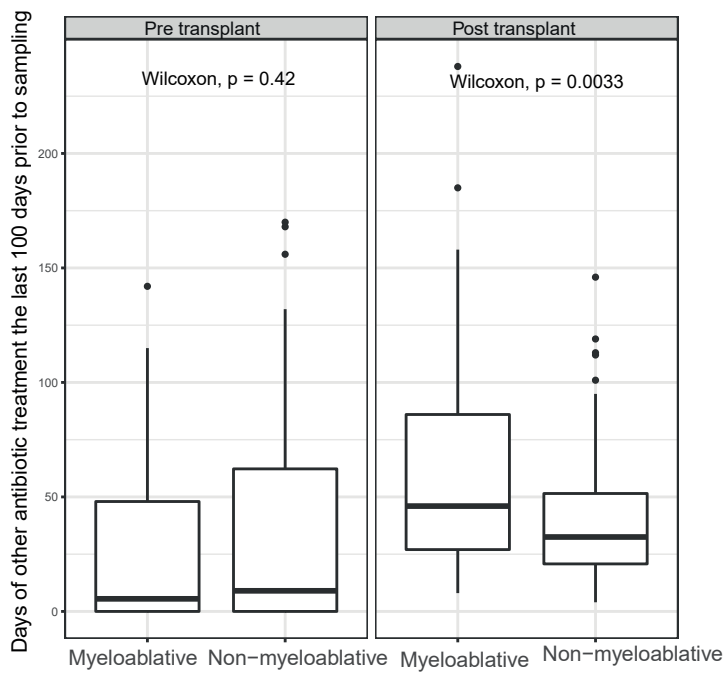

Figure S2

Supplement: Supplementary file 1 [file ijms-23-11115-s001.zip › S2.pdf]

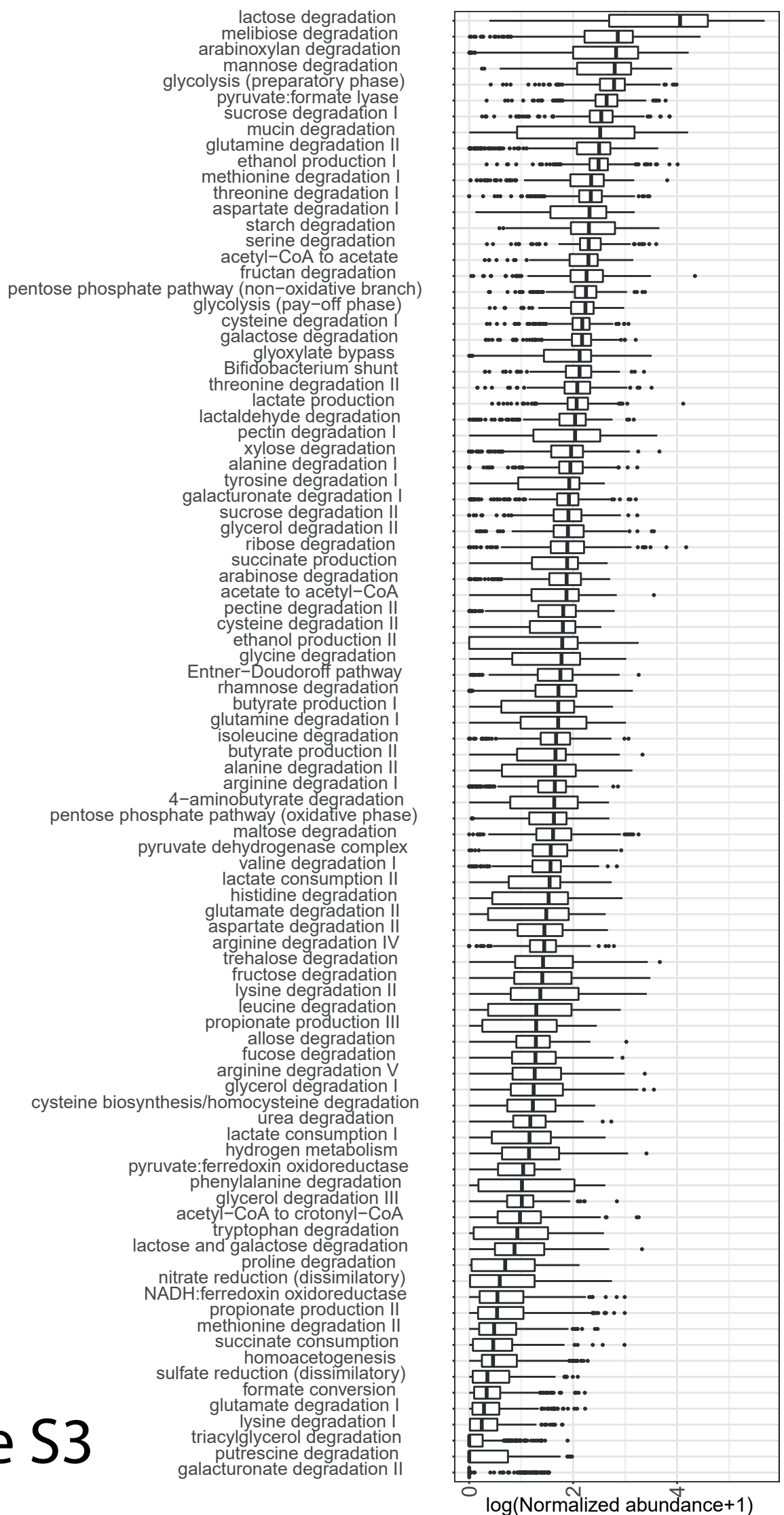

Figure S3

Supplement: Supplementary file 1 [file ijms-23-11115-s001.zip › S3.pdf]

**A)**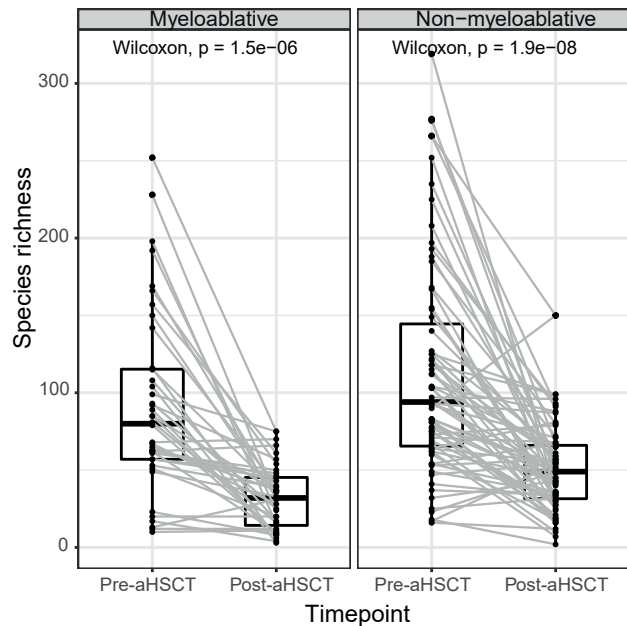**B)**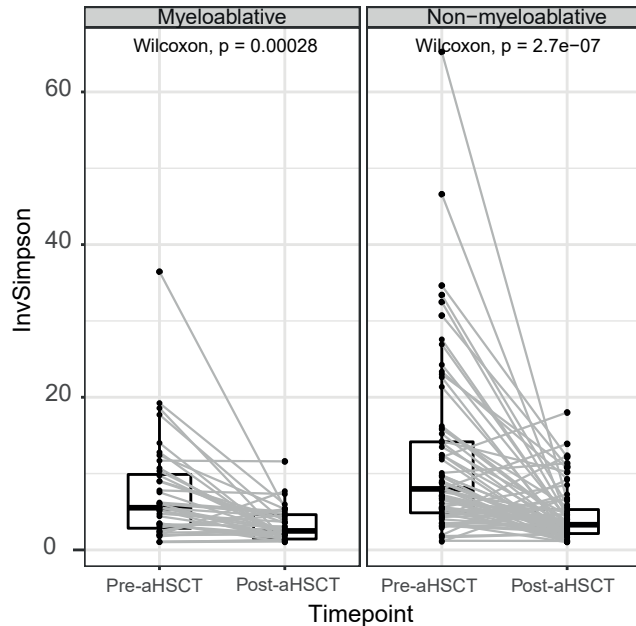**C)**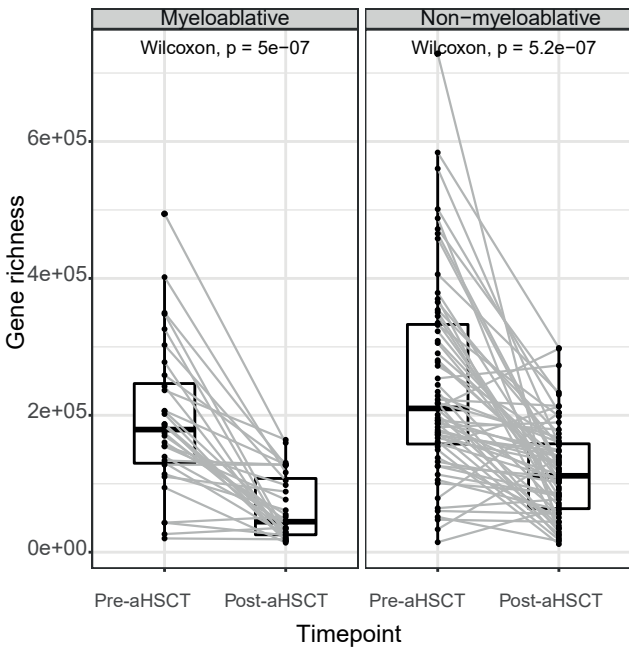**D)**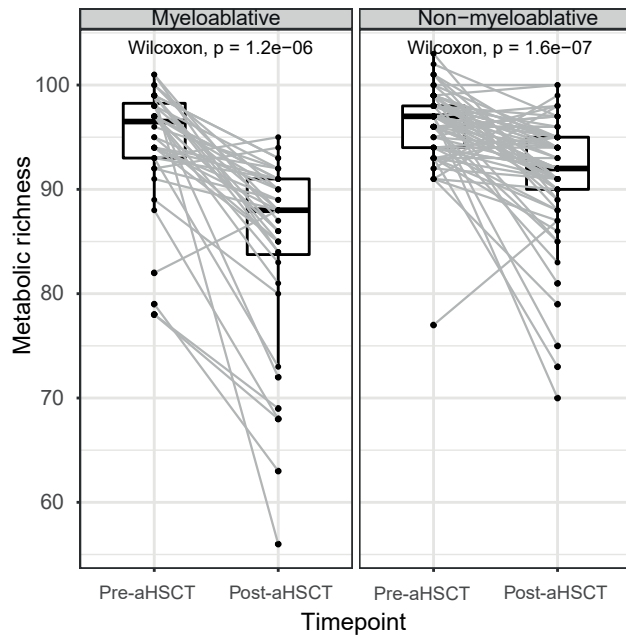

# Figure S4

Supplement: Supplementary file 1 [file ijms-23-11115-s001.zip › S4.pdf]

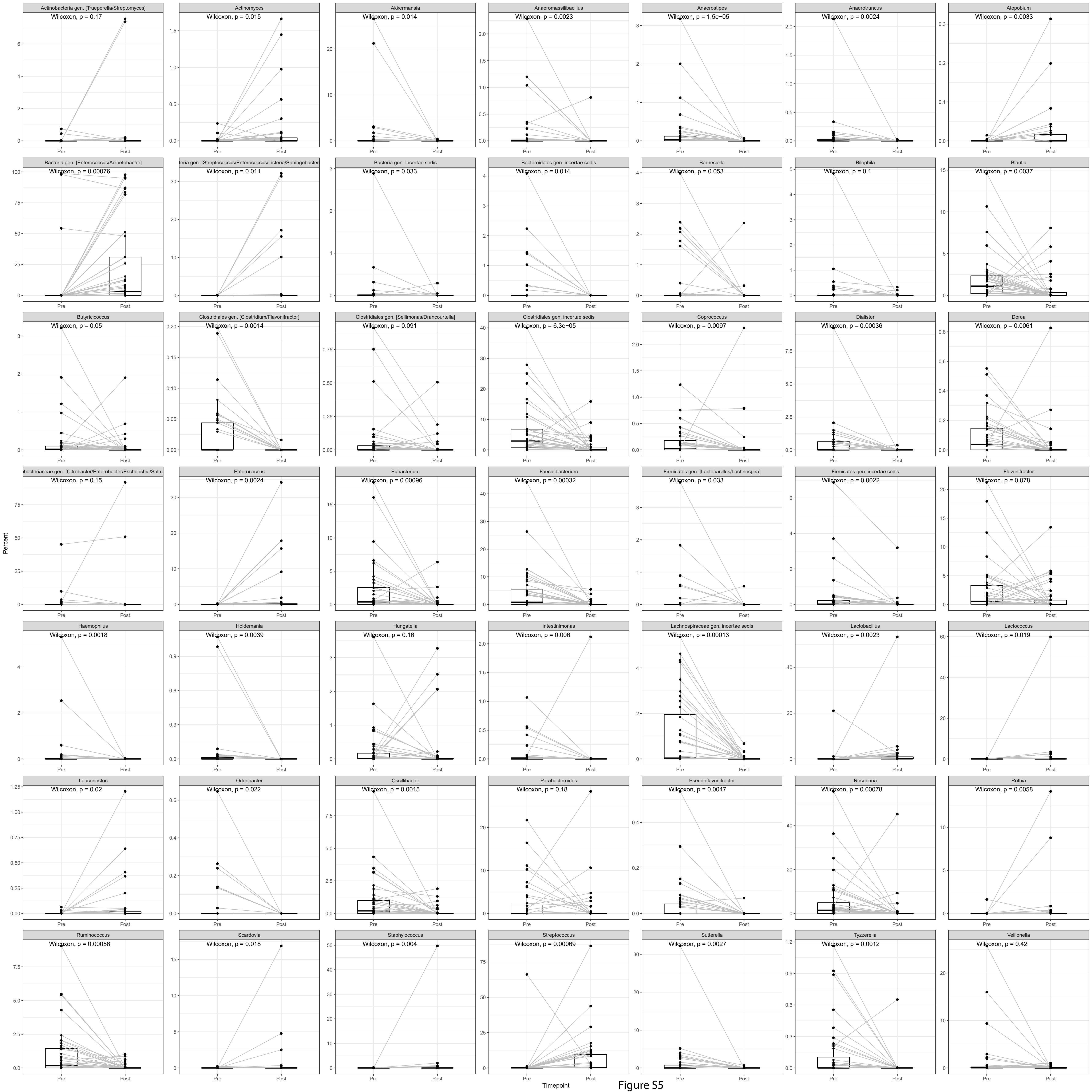

Supplement: Supplementary file 1 [file ijms-23-11115-s001.zip › S5.pdf]

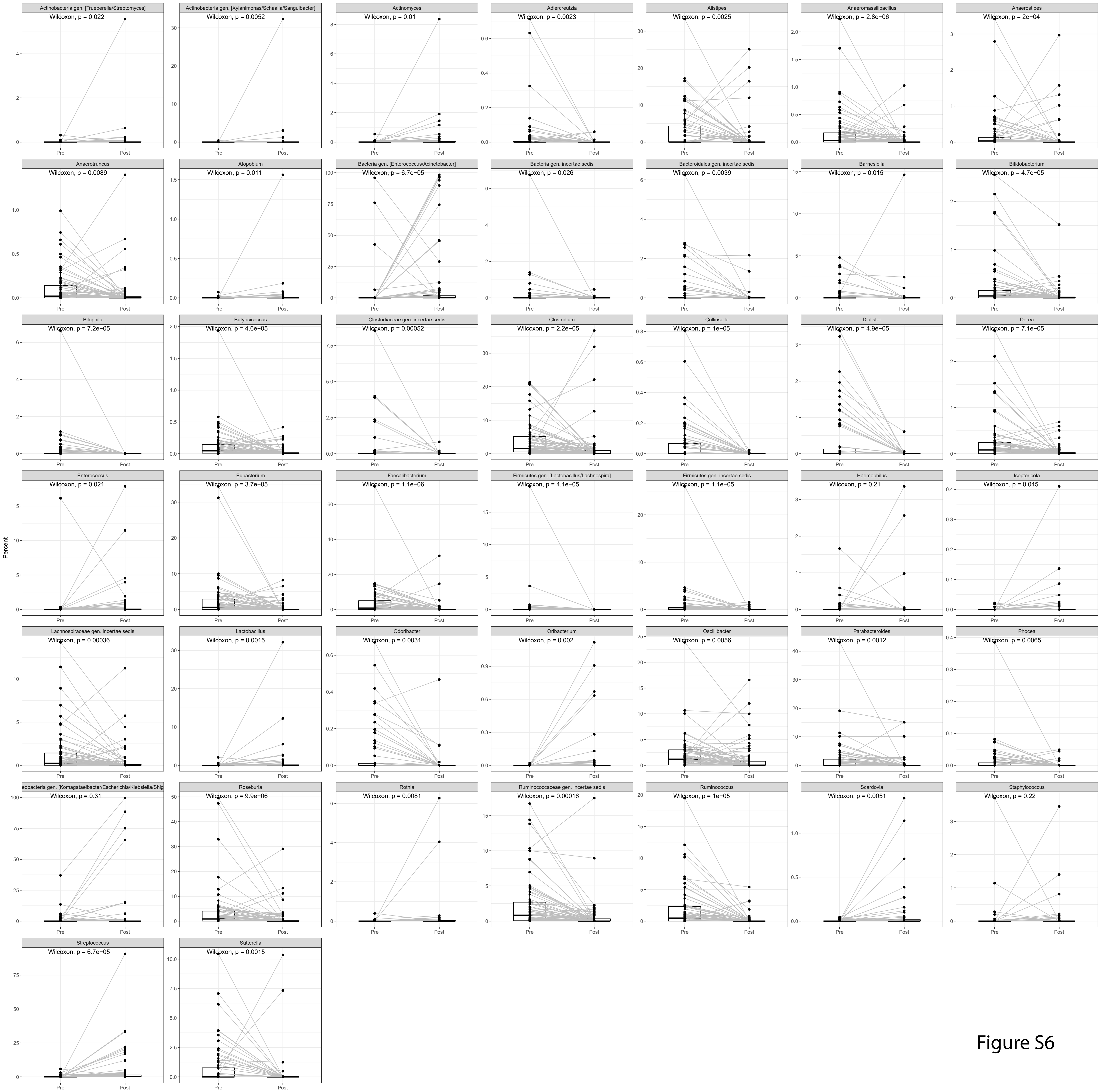

Figure S6

Supplement: Supplementary file 1 [file ijms-23-11115-s001.zip › S6.pdf]

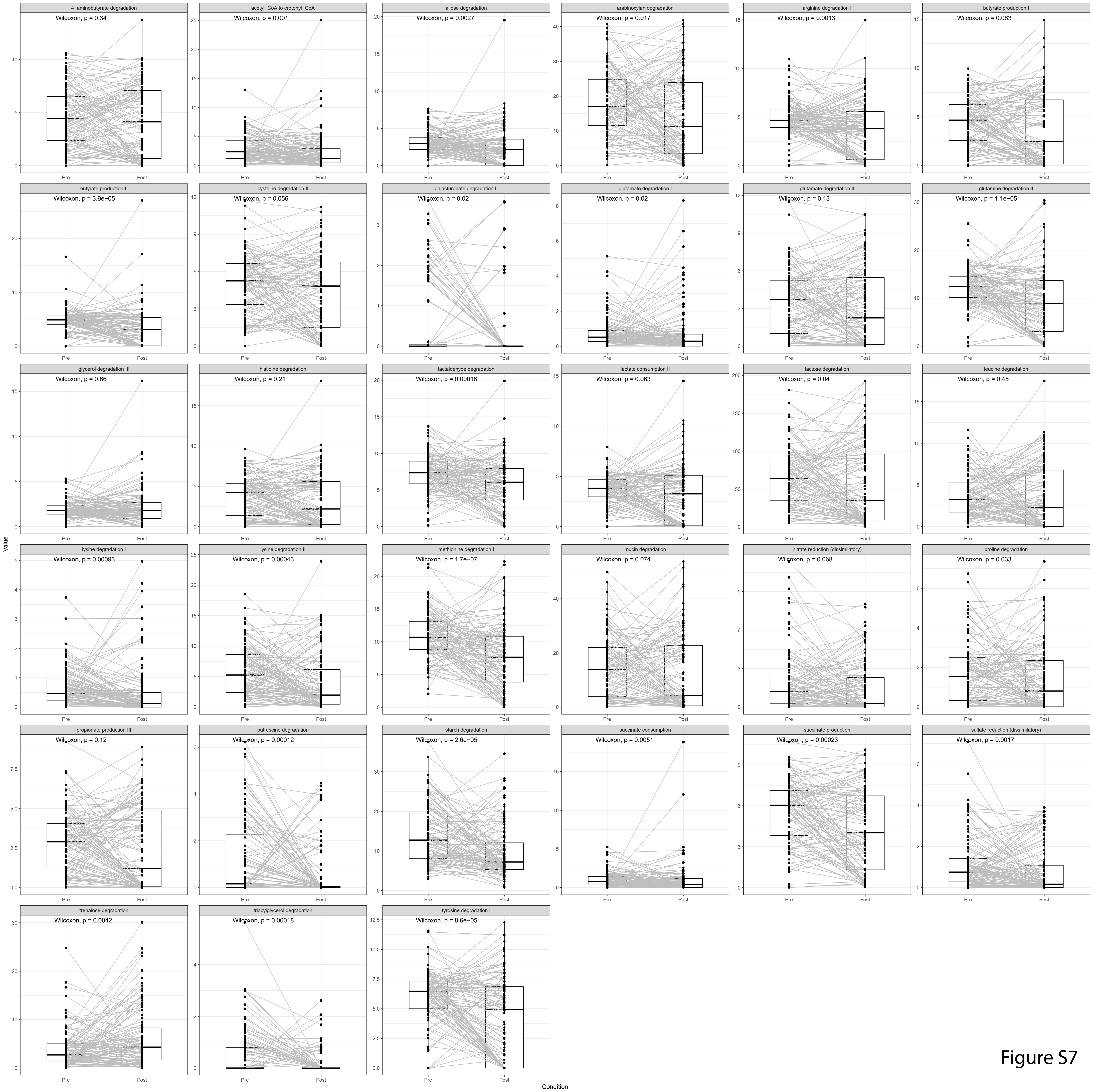

Figure S7

Supplement: Supplementary file 1 [file ijms-23-11115-s001.zip › S7.pdf]

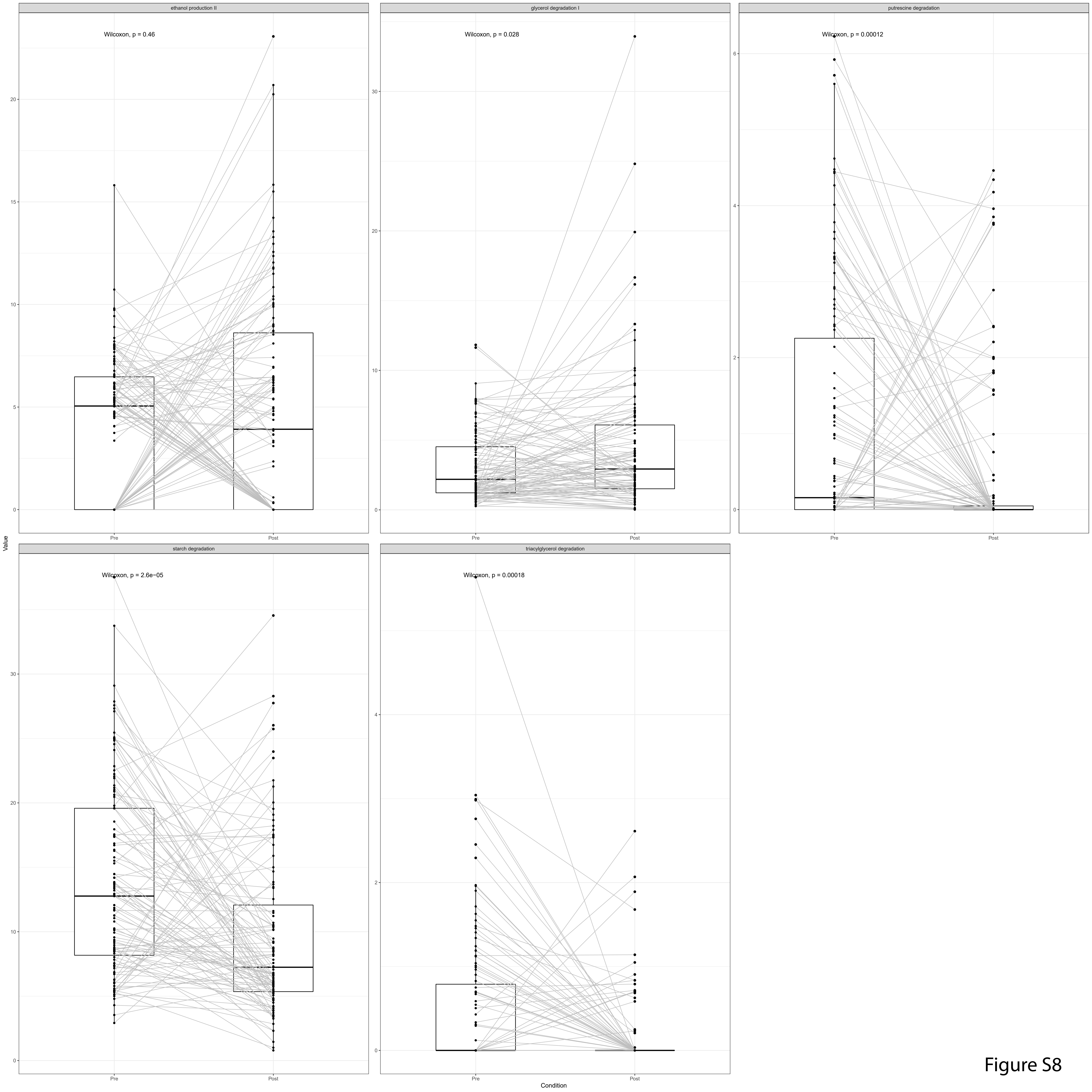

Supplement: Supplementary file 1 [file ijms-23-11115-s001.zip › S8.pdf]

A)

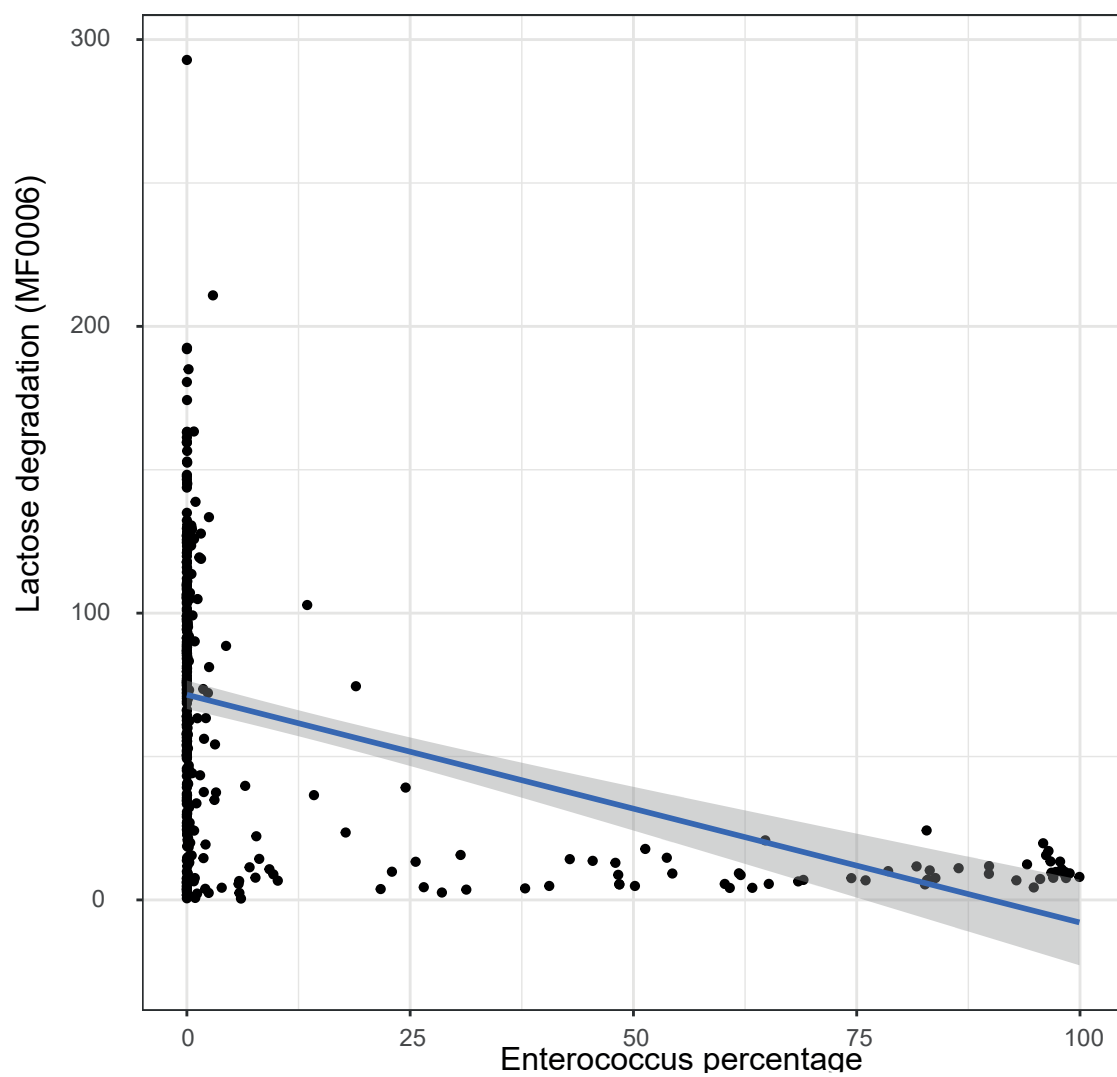

B)

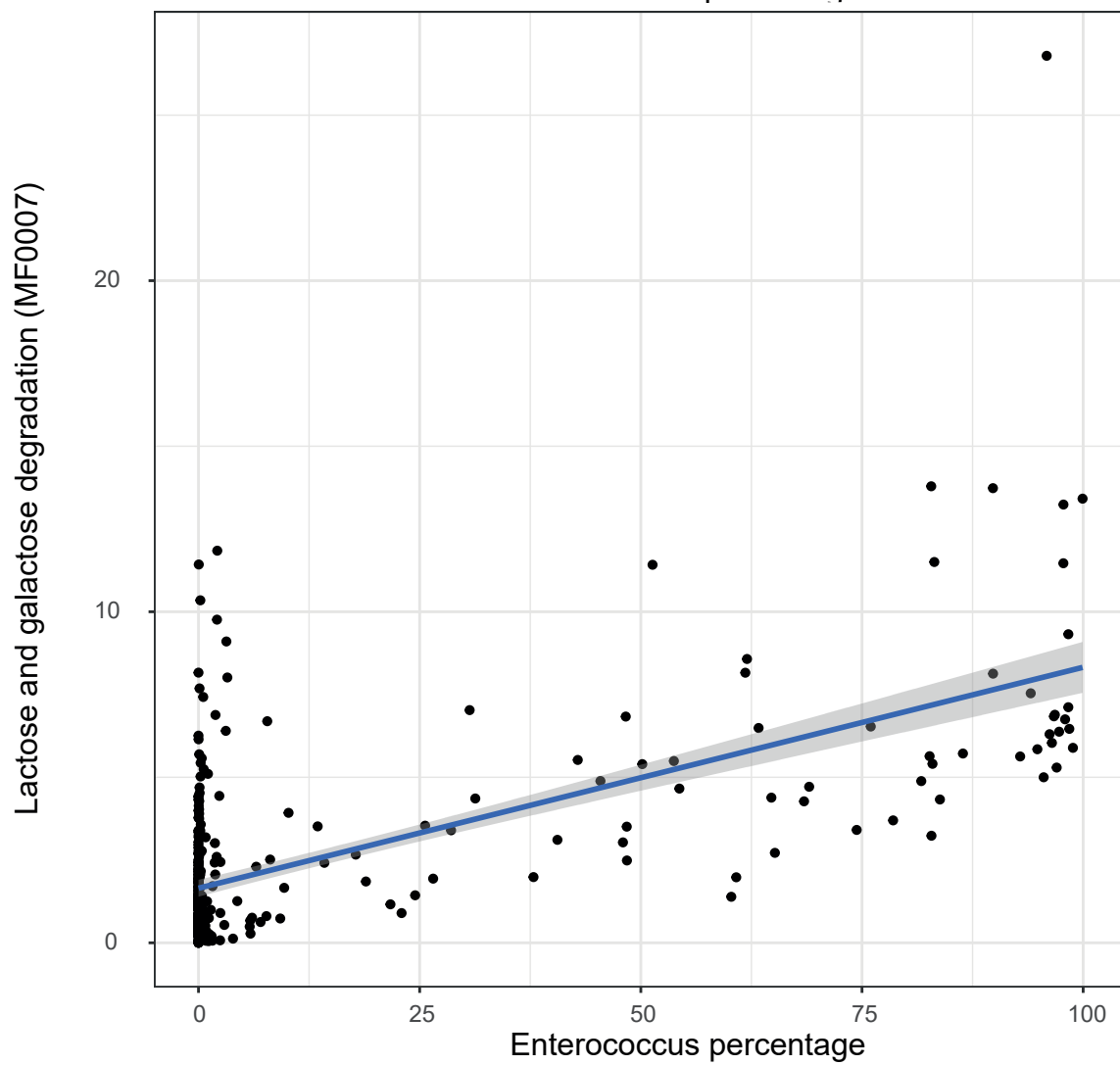

Figure S9

Supplement: Supplementary file 1 [file ijms-23-11115-s001.zip › S9.pdf]
